# Dual-Frequency Linear-to-Circular Polarization Converter for Ka-Band Applications

**DOI:** 10.3390/s22062187

**Published:** 2022-03-11

**Authors:** Francesco Greco, Emilio Arnieri

**Affiliations:** Millimeter-Wave Antennas and Integrated Circuits Laboratory (MAIC), DIMES University of Calabria, 87036 Arcavacata, Italy; f.greco@dimes.unical.it

**Keywords:** linear-to-circular polarization converter, dual-band, Ka-band

## Abstract

A dual-band linear-to-circular planar polarization converter based on a multilayer printed circuit board (PCB) is proposed and demonstrated. Each cell of the periodic surface is formed by six substrate layers separated by five foam spacers. The three top layers are identical and contain an ‘I’-type strip, while the three layers on the bottom side are realized with three identical Jerusalem crosses (JC). A linearly polarized (LP) wave tilted 45° relative to the *x*- and *y*-axis of the converter is used to illuminate the polarizer. In this configuration, right-handed circularly polarized (RHCP) waves are generated at the Ka-band while left-handed circularly polarized (LHCP) waves are generated at the K-band. An equivalent circuit model based on transmission lines is proposed and used to design the polarizer together with full-wave simulations. The simulated/measured axial ratio (AR) remains below 3 dB in the bands 19.4–21.8 GHz (12.5%) and 27.9–30.5 GHz (8.7%) with an insertion loss better than 0.5 dB.

## 1. Introduction

In recent years, wireless sensor networks (WSN) have attracted attention in the wireless communication domain for several applications such as medical and military surveillance, localization, smart homes, smart cities and monitoring. Circularly polarized (CP) antennas are often used in these applications [1,2,3,4] due to their ability to improve channel performance through alleviating multipath interference, low absorption losses and signal attenuation. CP can be generated in different ways depending on the kind of antenna. In this paper, circular polarization was generated by illuminating a planar polarization converter with a linearly polarized (LP) wave. With this approach, the converter and the antenna can be designed independently, thereby avoiding a complicated feeding network typically used in classical CP antennas. Planar polarizers can be divided into two categories: reflection-type circular polarizers [5,6,7,8] and transmission-type circular polarizers [9,10,11,12,13,14,15]. The transmission-type circular polarizers are more common and have been widely investigated in the literature.

The K/Ka frequency bands allocated in Europe for both civil and military satellite applications are 27.5–31 GHz (Ka-band) for the up-link case and 17.7–21.2 GHz (K band) for down-links. This type of link requires a high isolation between transmission (TX) and receiving (RX) channels that is obtained by assigning orthogonal polarizations to the two bands. As is well known, the use of a planar shared aperture antenna able to cover both bands can be a great advantage [16]. However, the single aperture configuration requires that the antenna, except when operating on the two TX and RX bands, has to be able to manage the dual polarization requirement. In this case planar Dual-band Linear to Circular Polarizers (LCPCs) are of much help. Dual band LCPCs are more difficult to design compared with single-band LCPCs. This is mainly due to mutual effects between the dual-band components. Some examples of circular polarizers have been presented in recent literature [13,17,18,19,20,21,22,23] for the dual-band case. While the solutions presented in [13,22,23] have a limited AR bandwidth at both bands, the configurations described in [17,18,19,20,21] have high insertion losses. In addition, several solutions available in the recent literature [21,22,23] are not able to provide orthogonal polarizations at the two bands.

In this paper, we propose a dual-band linear-to-circular planar polarization converter based on a multilayer printed circuit board (PCB). The proposed converter can generate right-handed circularly polarized (RHCP) waves at the Ka-band while left-handed circularly polarized (LHCP) waves are generated at the K-band. It should be noted that opposite polarization may be generated by flipping the polarizer with respect to one of its sides. The elementary cell is formed by six substrate layers separated by five foam spacers. An equivalent circuit model based on transmission lines is proposed using a first-pass design that is then refined by full-wave simulations.

The paper is organized as follows: Section 2 describes the working principle of the proposed elementary cell and introduces the equivalent circuit used in the design process; simulated and measured results are presented in Section 3 and Section 4, respectively; conclusions are finally discussed in Section 5.

## 2. Design of the Unit Cell

The selection of the most appropriate dual-band unit cell configuration must consider several aspects. In particular, high polarization isolation between the x-and y-polarizations should be guaranteed. This is achieved by adopting centrally connected elements such as classical or Jerusalem crosses [14,19,24,25,26] that have been widely used in these types of applications. Another important aspect is to guarantee a relative independence between the two bands.

**Figure 1:**
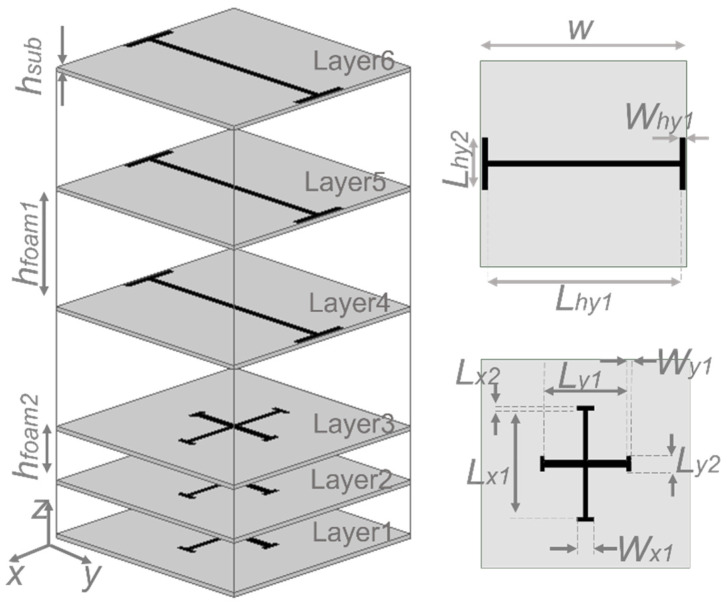
Configuration of the elementary cell.

Figure 1 shows the selected configuration. The cell is formed by six substrate layers separated by five foam spacers. The three layers on the bottom side (Layers #1, #2 and #3) are realized with three identical Jerusalem crosses (JC), the three top layers (#4, #5 and #6) are also identical and contain ‘I’-type strips. The three identical Jerusalem crosses are printed on 0.13 mm-thick (*hsub*) Rogers Ro3003 substrate layers separated by 1.5 mm of foam (*hfoam2)* spacer. The three identical ‘I’-type strips are printed on the same substrate and separated by 3.5 mm of foam (*hfoam1)* spacer. The combination of the ‘I’-type strips with Jerusalem crosses is used to achieve the desired dual band response.

A linearly polarized (LP) wave tilted 45° relative to the *x*- and *y*-axis of the converter is used to illuminate its surface. This wave can be seen as the superposition of two orthogonal components ($E_{x}^{i}$) and ($E_{y}^{i}$). Neglecting the mutual coupling between horizontal and the vertical components, the orthogonal components of the transmitted wave can be expressed in terms of the transmission coefficients of the unit cell for the *x* ($T_{x}$) and *y* polarizations ($T_{y}$) [24]:

(1) $$E_{x}^{t}=T_{x}E_{x}^{i};\;\;\;\;E_{y}^{t}=T_{y}E_{y}^{i}$$

where $T_{x}=\left|T_{x}\right|e^{i∡T_{x}}$ and $T_{y}=\left|T_{y}\right|e^{i∡T_{y}}$ are the transmission coefficients of the two orthogonal components. The circular polarization is generated imposing the following conditions in the band of interest:

(2) $$\left|T_{x}\right|=\left|T_{y}\right|$$

(3) $$∡T_{x}=∡T_{y}\pm 90{}^{\circ}$$

These conditions can be imposed using a single- or multi-layer Frequency Selective Surface (FSS) structure. However, the single-layer configuration typically suffers from narrow bandwidth due to the single resonant structure [27]. In contrast, the use of several metallization levels can increase the bandwidth of a generic FSS [28,29]. For this reason, a configuration with six dielectric layers separated by spacers of foam was employed in this work to ensure an optimal compromise between manufacturing complexity and AR bandwidth.

### Equivalent Circuit Model

In the literature, simplified equivalent models are largely used for the analysis of radiating structures Some of them use sophisticated analysis methods based on characteristic modes [30,31], whereas others resort to a more intuitive and experience-based construction of the circuit [32,33]. In the following, we adopt the second approach, leaving the first one to future developments.

**Figure 2:**
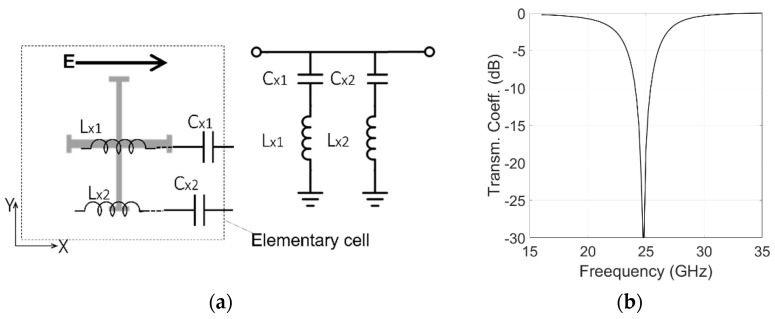
Equivalent circuit model of the Jerusalem cross for an x- polarized impinging wave. (**a**) Equivalent circuit; (**b**) transmission coefficient with Cx1 = 6.4 fF, Lx1 = 6.1 nH, Cx2 = 2.3 fF, Lx2 = 4.8 nH.

**Figure 3:**
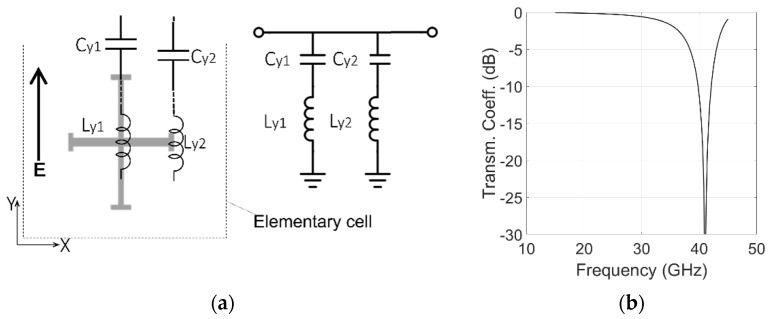
Equivalent circuit model of the Jerusalem cross for a y-polarized impinging wave. (**a**) Equivalent circuit; (**b**) transmission coefficient with Cy1 = 2.3 fF, Ly1 = 3.8 nH, Cy2 = 3.4 fF, Ly2 = 4.3 nH.

**Figure 4:**
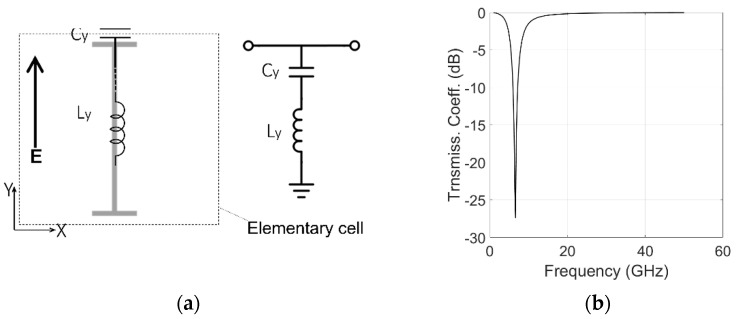
Equivalent circuit model of the ‘I’-type strip for a y- polarized impinging wave. (**a**) Equivalent circuit; (**b**) transmission coefficient with Cy = 72 fF, Ly = 6.3 nH.

Figure 2, Figure 3 and Figure 4 show the elements used in the elementary cell along with their equivalent circuit model. The ranges of physically realizable values of capacitances and inductances shown in the figures were established using Equations (5) and (6) of [34]. These closed-form equations have been used to estimate the feasible ranges from the minimum line/gap widths provided by the fabrication process. In our work, we used a cell periodicity of 7.15 mm. If a minimum realizable width of 50 µm for the conducting strips and the gaps is used in the equations, the feasible ranges for inductances and capacitances are 0.01–6.5 nH and 0.1–328 fF respectively.

The proposed configuration is regarded as a two-mode microwave circuit because the polarizer modifies the orthogonal and parallel incident fields differently. For an x-polarized wave incidence, the inductances and capacitances related to the horizontal branch of the cross are selected to be zero at a frequency between the two bands (Figure 2). In this manner, two passbands are generated, separated by the resonant stopband. For y-polarized waves, the vertical branch of the cross provides a zero at a high out-of-band frequency (Figure 3), offering a wide passband able to cover both bands. An ‘I’-type strip is added in a separate level to optimize magnitudes and phases of the y-polarized wave with minimum perturbation on the x-polarized wave. The ‘I’-type strip is sized to provide a pole at a frequency between the two bands (Figure 4).

**Figure 5:**
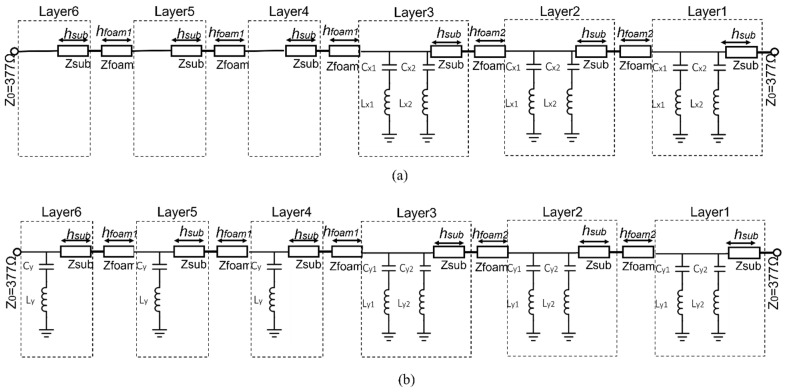
Equivalent circuit model of the multilayer polarizer for: (**a**) x-polarized input wave; (**b**) y-polarized input wave.

The three components described in Figure 2, Figure 3 and Figure 4 are then combined together to realize the configuration of the elementary cell shown in Figure 1. The equivalent circuit of the whole cell is shown for the x- and y-polarized waves in Figure 5a,b, respectively. In this model, each foam spacer between two layers is modelled with a *h_foam_* long transmission line with characteristic impedance $Z_{foam}=$ $Z_{0}=377\Omega$ and propagation constant $\beta_{0}=\omega /c$, where *c* is the speed of light. Each dielectric substrate is modelled with a *h_sub_* long transmission line with characteristic impedance $Z_{sub}=Z_{0}/\sqrt{\varepsilon_{r}}$ and $\beta_{sub}=\beta_{0}\sqrt{\varepsilon_{r}}$.

The circuits shown in Figure 5 can be used for a further optimization of the parameters using Keysight Advanced Design System (ADS) to satisfy the objective of maximizing the −3 dB AR bandwidths at Rx and Tx band respectively.

## 3. Simulated Results

The initial dimensions of the converter were obtained using the proposed equivalent circuit model. These values have then been used as a starting point for a final optimization of the unit cell in Ansys High Frequency Simulation Software (HFSS) [35].

### 3.1. Equivalent Circuit Simulations

**Figure 6:**
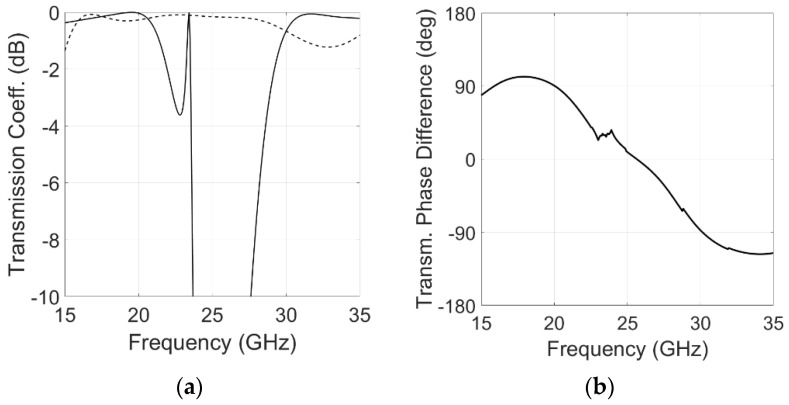
Simulated transmission coefficients of the equivalent circuit of the proposed converter. (**a**) Module of the transmission coefficient: Continuous line, x-polarization; dashed line, y-polarization. (**b**) Transmission coefficient phase difference.

According to Equations (2) and (3), the two equivalent circuits shown in Figure 5, are used to realize a quadrature phase shift between the x and y components of the incident electric field, providing a 90° phase difference between the transmission coefficients of the two components and minimizing the insertion loss. Both circuits are matched to the equivalent impedance (Z0 = 377 Ω). The capacitances and inductances values shown in Figure 2, Figure 3 and Figure 4 are used in the equivalent circuit model of the multilayer polarizer shown in Figure 5. These values were then slightly modified in a final optimization. The following values were obtained: Cx1 = 6.3 fF, Lx1 = 6.5 nH, Cx2 = 2.3 fF, Lx2 = 4.8 nH, Cy1 = 2.3 fF, Ly1 = 3.8 nH, Cy2 = 3.3 nH, Ly2 = 4.0 nH, Cy = 72 fF, Ly = 6.3 nH, *hfoam*1 = 3.5 mm, *hfoam*2 = 1.5 mm, *hsub* = 0.13 mm. To have circular polarization, both Conditions (2) and (3) have to be fulfilled by the two transmission coefficients of the equivalent circuits. Modules for both polarizations are shown in Figure 6a. Condition (2), $\left|T_{x}\right|=\left|T_{y}\right|$, is fulfilled in the two bands of interest. Figure 6b shows the transmission phase difference. Condition (3) is fulfilled in the TX band with the ‘+’ sign generating an LHCP polarization. In contrast, RHCP is generated around 30 GHz where the condition is fulfilled with the ‘−’ sign.

### 3.2. Full Wave Simulations

**Figure 7:**
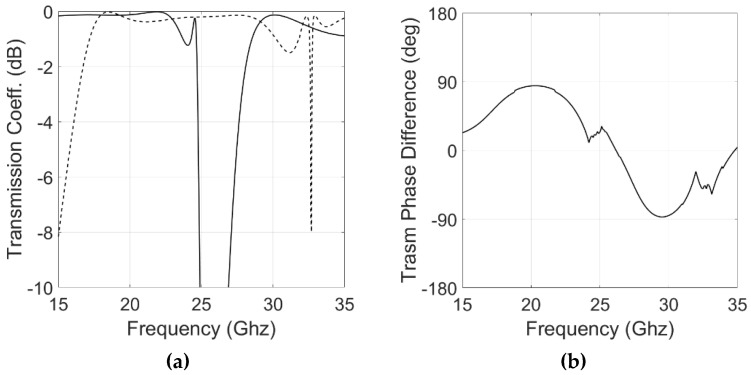

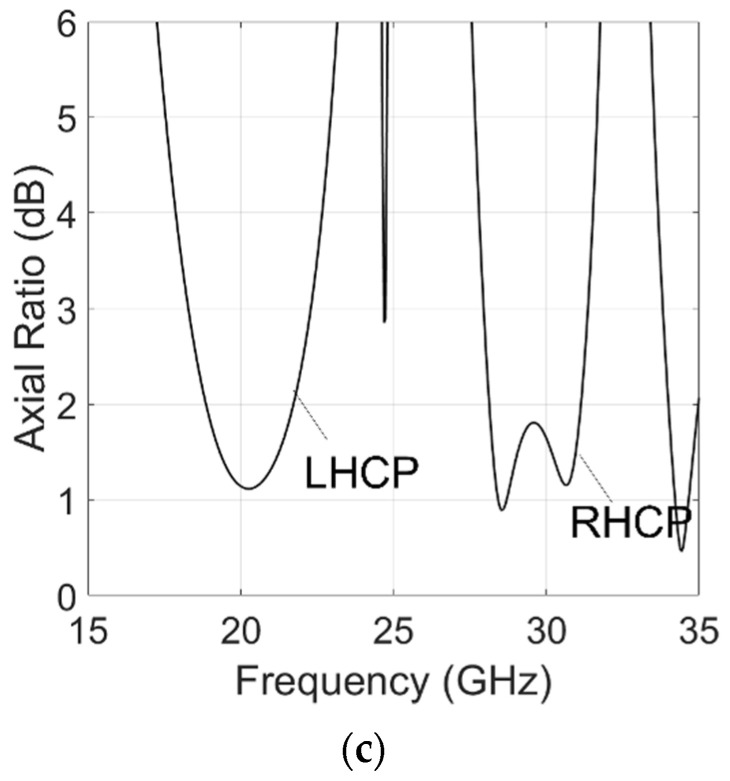
HFSS simulated results of the elementary cell. (**a**) The module of the transmission coefficient: Continuous line, x-polarization; dashed line, y-polarization. (**b**) Transmission coefficient phase difference. (**c**) Axial Ratio.

The proposed equivalent circuit model was used to obtain a preliminary estimation of the converter geometrical dimensions; however, a final optimization with a full-wave simulator was needed to perform a fine-tuning of the analytic solution. Using the capacitances and inductances obtained with the circuit model, it was possible to estimate the initial physical dimensions of the converter using Equations (5) and (6) of [34]. An HFSS model was created to perform the optimization of the cell. The final optimal values are (see Figure 1): *W* = 7.15 mm, *Lhy*1 = 6.65 mm, *Why*1 = 0.2 mm, *Lhy*2 = 1.8 mm, *Ly*1 = 2.8 mm, *Wy*1 = 0.15 mm, *Ly*2 = 0.525 mm, *Lx*1 = 3.66 mm, *Lx*2 = 0.15 mm, *hfoam*1 = 3.5 mm, *hfoam*2 = 1.5 mm, *hsub* = 0.13 mm. HFSS simulated results are shown in Figure 7 and compare well with the response predicted by the equivalent circuit model.

Figure 7c shows the simulated Axial Ratio (AR). −3 dB bandwidths reach 20.5% (18.2–22.3 GHz) and 12% (28–31.5 GHz) in the lower and upper bands, respectively, which cover a large part of the operation bandwidths for K/Ka-band satellite communication.

## 4. Measured Results

**Figure 8:**
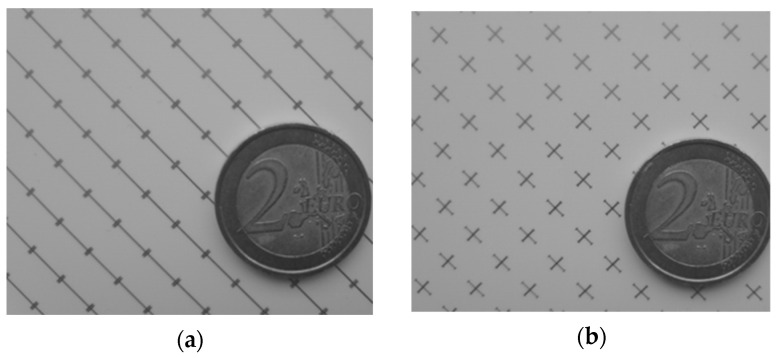
Manufactured prototype: (**a**) Top side; (**b**) bottom side.

**Figure 9:**
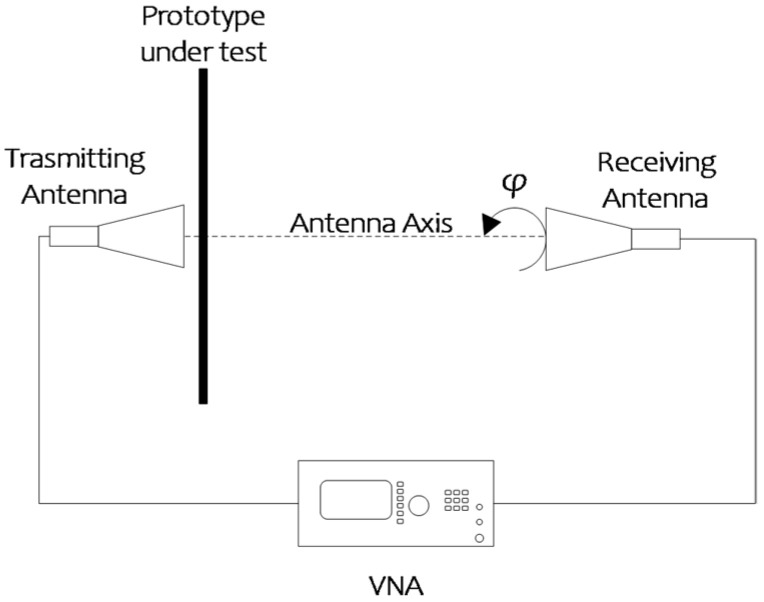
Block diagram of the measurement setup.

The proposed dual-band linear-to-circular polarization converter was fabricated using a standard PCB manufacturing process and it is shown in Figure 8. The six Rogers Ro3003 substrate layers are interleaved with five foils of Rohacell foam with the following thickness: *hfoam*1 = 3.5 mm and *hfoam*2 = 1.5 mm (Figure 1). 38 µm of glue was necessary between the dielectric substrates and the foam. It should be noted that converter performances are affected by the glue layers. In particular, a small frequency shift and bandwidth reduction were observed in simulations. The characterization of the prototype was realized using a measurement setup similar to the one used in [13] and is shown in Figure 9.

The prototype was placed between two standard horns used as transmitting and receiving antennas. The two antennas were connected to a vector analyser (VNA) for S-parameters measurement. The accuracy of the phase measurement can be severely limited by the test environment. For this reason, a method based on amplitude-only measurements was used to evaluate the Axial Ratio. During the test, the receiving antenna was rotated around its axis at *φ* = 0°, 90°, 45° and 135° (see Figure 9). The four linear polarization amplitudes *E_1_*, *E_2_*, *E_3_*, *E_4_* acquired during the rotation were used in relations (11) and (12) of [36] to calculate the AR.

**Figure 10:**
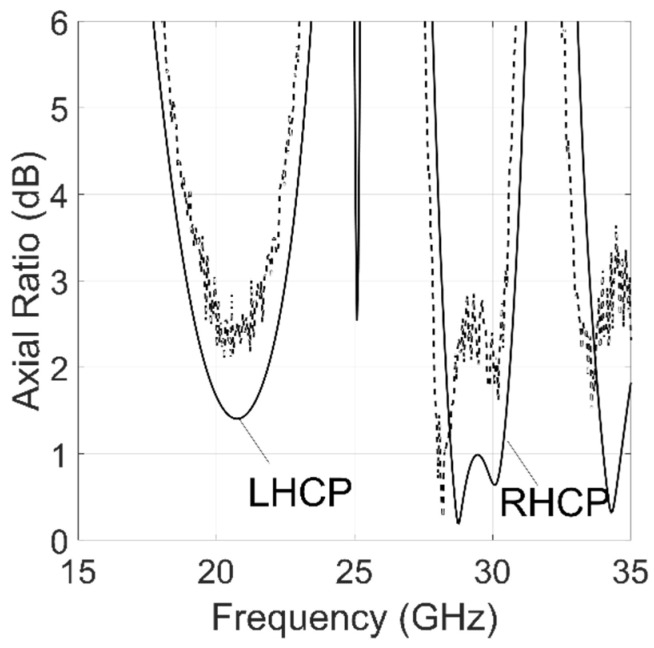
Simulated (continuous line) and measured (dashed line) ARs of the proposed converter. Glue layers have been included in the simulation.

The measured axial ratios are compared with the simulations in Figure 10. The measurements show close agreement with the simulation values if the glue layers are included in the HFSS model. A bandwidth reduction and a limited frequency shift were observed in the experimental data and are attributed to fabrication tolerances. The AR remains below 3 dB in the bands 19.4–21.8 GHz (12.5%) and 27.9–30.5 GHz (8.7%). The maximum measured insertion loss is 0.5 dB and 0.4 dB at the lower and upper frequency respectively.

Table 1 shows the performance of the dual-band converter proposed in this paper compared with other solutions presented in the recent literature. The proposed design compares well with other cases in terms of both bandwidth and insertion losses.

## 5. Conclusions

This paper presents the realization and the measurement of a dual-band linear-to-circular polarization converter based on a multilayer printed circuit board (PCB). The converter consists of six substrate layers separated by five foam spacers. The three top layers are identical and contain an ‘I’-type strip while the three layers on the bottom side are realized with three identical Jerusalem crosses (JC). An equivalent transmission line circuit model was used for the preliminary design of the unit cell which provides an initial estimate for further full-wave optimization. The output wave was converted to an LHCP signal in the lower band, and an RHCP signal in the upper band when transmitted through the proposed surface. A prototype was fabricated and measured. A close agreement was observed between simulated and measured results. The measured AR bandwidth was 12.5% and 8.7% in the K and Ka band respectively, with a maximum insertion loss of 0.5 dB.

## Figures and Tables

**Table 1:** Performances comparison among dual-band LCPCs.

Ref.	Technology	Center Freq. (GHz)	Insertion Loss (dB)	AR Bandwidth	Cell Size	Orthogonal Polarizations
[13]	Patch and a split ring	19.95, 29.75	<1, <2	5%, 7%	0.35 λ_1_	
[20]	Split ring resonators	8.8, 10.3	6	-	0.1 λ_1_	Yes
[22]	Split ring resonators	15.1, 16.5	10, 5	11.8%, 6.9%	0.4 λ_1_	No
[23]	Modified Jerusalem Cross	19.6, 29	0.6, 0.6	4%, 2.7%	-	No
[21]	Split ring resonators	7.6, 13	1.5	31%, 13.8%	0.22 λ_1_	No
[18]	Chiral Metamaterial	5.1,6.4	1.6, 4	-	0.26 λ_1_	Yes
[19]	Jerusalem Cross	18.5, 29	2, 0.8	29%, 12%	0.25 λ_1_	Yes
[17]	Chiral Metamaterial	9.77, 11.84	1.6, 4	-	0.214 λ_1_	Yes
This Work	Jerusalem Cross/’I’-type strips	19.95, 29.75	0.5, 0.4	12.5%, 8.7%	0.47 λ_1_	Yes

## Data Availability

The study did not report any data.
